# Determination of CAR T cell metabolism in an optimized protocol

**DOI:** 10.3389/fbioe.2023.1207576

**Published:** 2023-06-20

**Authors:** Sandy Joaquina, Christopher Forcados, Benjamin Caulier, Else Marit Inderberg, Sébastien Wälchli

**Affiliations:** ^1^ Translational Research Unit, Department of Cellular Therapy, Oslo University Hospital, Oslo, Norway; ^2^ Center for Cancer Cell Reprogramming (CanCell), Institute for Clinical Medicine, Faculty of Medicine, University of Oslo, Oslo, Norway; ^3^ Department of Molecular Cell Biology, Institute for Cancer Research, Oslo University Hospital, Oslo, Norway

**Keywords:** CAR T cell, Seahorse XF96, metabolim, immunotherapy, cancer therapy

## Abstract

Adoptive transfer of T cells modified to express chimeric antigenic receptors (CAR) has emerged as a solution to cure refractory malignancies. However, although CAR T cell treatment of haematological cancers has now shown impressive improvement in outcome, solid tumours have been more challenging to control. The latter type is protected by a strong tumour microenvironment (TME) which might impact cellular therapeutic treatments. Indeed, the milieu around the tumour can become particularly inhibitory to T cells by directly affecting their metabolism. Consequently, the therapeutic cells become physically impeded before being able to attack the tumour. It is therefore extremely important to understand the mechanism behind this metabolic break in order to develop TME-resistant CAR T cells. Historically, the measurement of cellular metabolism has been performed at a low throughput which only permitted a limited number of measurements. However, this has been changed by the introduction of real-time technologies which have lately become more popular to study CAR T cell quality. Unfortunately, the published protocols lack uniformity and their interpretation become confusing. We herein tested the essential parameters to perform a metabolic study on CAR T cells and propose a check list of factors that should be set in order to draw sound conclusion.

## 1 Introduction

For the past two decades, chimeric antigen receptor (CAR)-modified T cells have emerged as an innovative and promising therapy for refractory cancers. This strategy involves genetic modification of T cells in order to provide them the ability to get activated through specific recognition of antigens expressed at the surface of tumour cells. The most exploited target is indisputably CD19, a B-cell lineage marker, which arose as an excellent CAR target reaching the approval of both US (FDA) and European (EMA) agencies (Neelapu et al., 2017; Maude et al., 2018). In 2021, further, development made four new promising CD19CARs available, Breyanzi, Yescarta, Kymriah and Tecartus, as well as a CAR targeting B cell maturation antigen (BCMA) in myeloma, Abecma and Carvykti (CAR T Cells, 2023). In contrast, solid tumours patients have so far benefited less from CAR therapy despite the high number of clinical trials run worldwide (Sterner and Sterner, 2021; Chen et al., 2022; Del Bufalo et al., 2023). Indeed, several challenges hampering CAR T cell efficacy have yet to be overcome and include i) poor target recognition, ii) low tumour infiltration, iii) suboptimal T cell activation and iv) lack of persistence on site (Guedan et al., 2019; Wagner et al., 2020; Marofi et al., 2021). Recent studies have highlighted that most of these barriers are, in part, related to the immunosuppressive tumour microenvironment (TME) and the overall fitness of the CAR T cell product (RialSaborido et al., 2022). To counteract these effects, the co-expression of a CAR and metabolic enzymes such as the argininosuccinate synthase and the ornithine transcarbamylase have been investigated and shown to improve CAR T cell persistence and efficacy upon infusion (Fultang et al., 2020). Accordingly, numerous studies have linked efficient metabolic pathways to competent CAR T cell functions, as reviewed in Forcados et al., (2022); Peng et al., (2023). Effector T cells, ready to produce cytokines and kill, favour glycolysis over oxidative phosphorylation (OXPHOS) for rapid activation and function. In contrast, memory T cells, which are maintained long term and less metabolically active, but can quickly differentiate into effector cells for sustained immune responses, favour OXPHOS over glycolysis (Rostamian et al., 2021). Recent studies have led to broaden the metabolic strategies also on the use of epigenetics for metabolism modulation and on reprogramming fatty acid metabolism for CAR T cells (Huang et al., 2022; Ito and Kagoya, 2022). Therefore, studying the metabolic fitness of CAR T cells should help to better predict the efficacy of the therapy including its resistance to the inhibitory TME.

**FIGURE 1:**
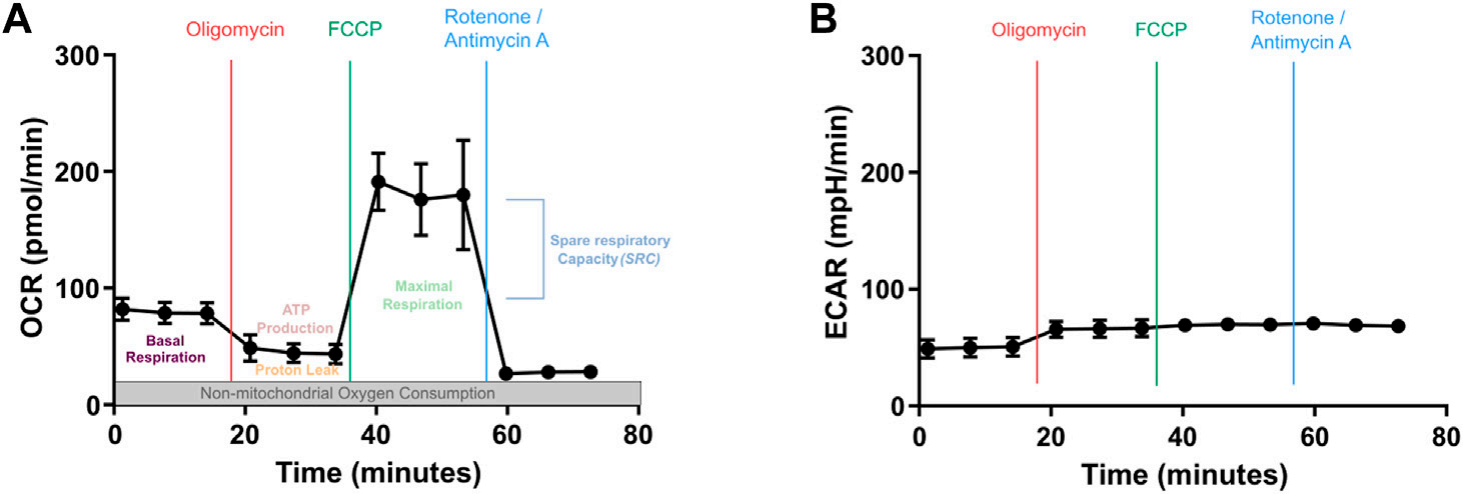
Mitochondrial metabolic analysis by Seahorse XF Cell Mito Stress Kit. **(A)** Oxygen consumption rate (OCR) profile and **(B)** extracellular acidification rate (ECAR) profiles during the same metabolic monitoring. Basal OCR measurement is performed over the three first time points. This is followed by the measurement of OCR after sequential addition of Oligomycin (inhibits ATP synthesis, which stops the electron transport and blocks OXPHOS), Carbonyl cyanide-p-trifluoromethoxyphenylhydrazone (FCCP, proton uncoupler that mimics a sudden high need in energy, hence will show the maximum respiration rate) and Rotenone/Antimycin A (strong blockers of electron transport, lead to complete inhibition of OXPHOS). Three measures/drug are performed.

In this report, we compare different key parameters for capturing steady state metabolism of CAR T cells using the Seahorse XF analyzer, which allows the assessment of cellular functions such as mitochondrial respiration and glycolysis. We focus on the study of mitochondria using the Mito Stress assay, which allows a precise examination of OXPHOS in real-time by measuring changes in the oxygen consumption rate (OCR) imputable to Adenosine triphosphate (ATP) renewal, proton leakage and electron transfer capacity. In addition, it can measure glycolysis determined by the rate of extracellular acidification (ECAR) (Figure 1). Despite the increased use of this technology in the evaluation of CAR T cell therapy candidates several protocols found in the literature may not always be optimal (Forcados et al., 2022). The aim in the current study was therefore to compare different T cell and drug concentrations to provide an optimized protocol for the evaluation of CAR T cell metabolism.

## 2 Materials and equipment

### 2.1 Cells and reagents

PBMCs were isolated from healthy donors by density gradient centrifugation from buffy coats obtained from the Oslo blood bank (Ethical approval 2019/121). Complete culture medium was made with X-VIVO 15 (Lonza, Basel, Switzerland) supplemented with 5% of serum replacement (Thermo Fischer Scientific, Waltham, MA, United States) and 100 U/mL recombinant human IL-2 (Proleukin, Prometheus Laboratories Inc., San Diego, CA, United States). The CD19CAR retroviral vector (pMP71) expressed a second generation FMC63-based anti-CD19 CAR designed as follows: CD8h-CD8tm-4-1BB-CD3z, its cloning and production was previously described (Köksal et al., 2019).

### 2.2 Retroviral transduction of human T cells

T cells were activated from PBMCs following this protocol: PBMCs were resuspended in complete X-VIVO 15 and activated for 2 days at 1 × 10^6^ cells/well in a 24-well plate pre-coated with anti-CD3 and anti-CD28 antibodies (each at 1 μg/mL, clones: OKT-3 and CD28.3, respectively, Thermo Fisher Scientific). Afterwards, T cells were spinoculated with retroviral supernatants (previously prepared as described in Köksal et al., (Hakan et al., 2019)) at 32°C at 750 × g for 60 min in a non-tissue culture treated 24-well culture non-treated plate (Nunc A/S, Roskilde, Denmark) pre-coated with retronectin (50 μg/mL, Takara Bio. Inc., Shiga, Japan). The spinoculation was repeated once more the following day with fresh medium and retroviral supernatant. Then, T cells were transferred to new flasks and maintained in culture. Transduction efficiency was verified after 5–10 days by staining human CD19 (20-291) protein, Fc Tag (Accro Biosystem, Newark, DE, United States) and Allophycocyanin (APC) AffiniPure F(ab′)₂ Fragment Goat Anti-Human IgG, Fcγ fragment specific (ThermoFisher, Waltham, MA United States) assessed by Flow Cytometry (Köksal et al., 2019; Köksal et al., 2020; Pierre et al., 2021).

The T cells were kept at a concentration of 1 × 10^6^ cells/mL throughout the culture period, with fresh medium added every second day. The cells were used on day 10 after transduction.

### 2.3 Seahorse mito stress assay

The following protocol takes into account the suppliers’ recommendations. The day before the ex-periment, the sensor cartridge was rehydrated in the Utility plate with 75 µL of sterile water/well and kept at 37°C in a CO2-free incubator. The cell culture microplates were placed in a non-CO2 incubator at 37°C and the cells were brought to a concentration of 1 × 10^6^ cells/mL with the addition of fresh medium. On the day of the experiment, the water was replaced with 75 µL of calibrant for at least 1 h before starting the assay.

Cell culture microplates were first coated with 22.4 µg/mL of Cell-Tak (Corning Inc., Corning, NY, United States) for 20 min at room temperature. After two washings with 200 µL of distilled water per well, the culture plate was seeded with 50 µL of T cells at the indicated concentration. T cells were firstly washed in XF medium supplemented with 5.5 mM glucose, 1 mM sodium pyruvate and 2 mM L-glutamine before being resuspended in the same medium at the correct concentration. Then, after a 20 min incubation at 37°C in a non-CO2 incubator, 130 µL of complete XF medium maintained at 37°C in a non-CO2 incubator was carefully added to each well before continued incubation for at least 20 min at 37°C in a CO2-free incubator. The medium was gently added on the side of the well in order to not disturb the monolayer. The conservation of the cell monolayer was controlled by microscopy upon addition of 130 µL of medium (data not shown). In the meantime, Oligomycin, FCCP, and Rotenone/Antimycin A (Seahorse XF Cell Mito Stress Test kit, Agilent Technologies, Santa Clara, CA, United States) were resuspended in complete XF medium and then loaded into their respective ports. The media was kept at 37°C, in a water bath, throughout the preparation. Importantly, each condition was performed in sextuplicate and 6 wells of the cell culture microplate were always kept to measure the background containing only complete XF media. Finally, the assay was started on a Seahorse XF96 analyzer (Agilent Technologies) with a first calibration of the cartridge for 20 min before loading the cell plate. The XF Mito Stress Test module was selected on the software (Wave, Agilent Technologies). All the experiments were carried out using two different transductions. A single, large batch of T cells was expanded for 10 days, frozen at −80°C and used throughout the study to ensure experimental reproducibility. The cells were thawed 24–48 h before a Seahorse run to ensure a full T cell metabolism recovery (Welters et al., 2012; Mander et al., 2010).

### 2.4 T cells stimulation

T cells were seeded in a 96-well plate pre-coated overnight at 4°C with human CD19 protein (from amino acid 20-291) with Fc Tag (at 1/50, ACROBiosystems) or with a mix of anti-CD3 and anti-CD28 antibodies (each at 1 μg/mL, clones: OKT-3 and CD28.3, respectively, Thermo Fischer Scientific). T cells were seeded at 2 × 106 cells/well. After 2 h on the coating, the cells were washed twice in complete XF medium before being transferred to Cell culture microplates coated with Cell-Tak.

### 2.5 Microscopy

The Cell-Tak-coated cell culture microplate seeded with T cells was placed in a microscope at 37°C (Incucyte S3, Essen Bioscience Ltd., Ann Arbor, MI, United States). Four images (phase) were taken per well at a ×10 objective after the addition of the cells, before and after the incubations described above, prior to placing the microplate in the XF96 analyzer.

### 2.6 Statistics

Data were exported from Wave (Wave software, Agilent Technologies), and analyzed with GraphPad (GraphPad software, San Diego, CA, United States). The results are presented as the mean ± standard deviation (SD). Two-way and one-way Anova multiple comparisons tests were used.

## 3 Results

### 3.1 Optimization of T cell concentration

**FIGURE 2:**
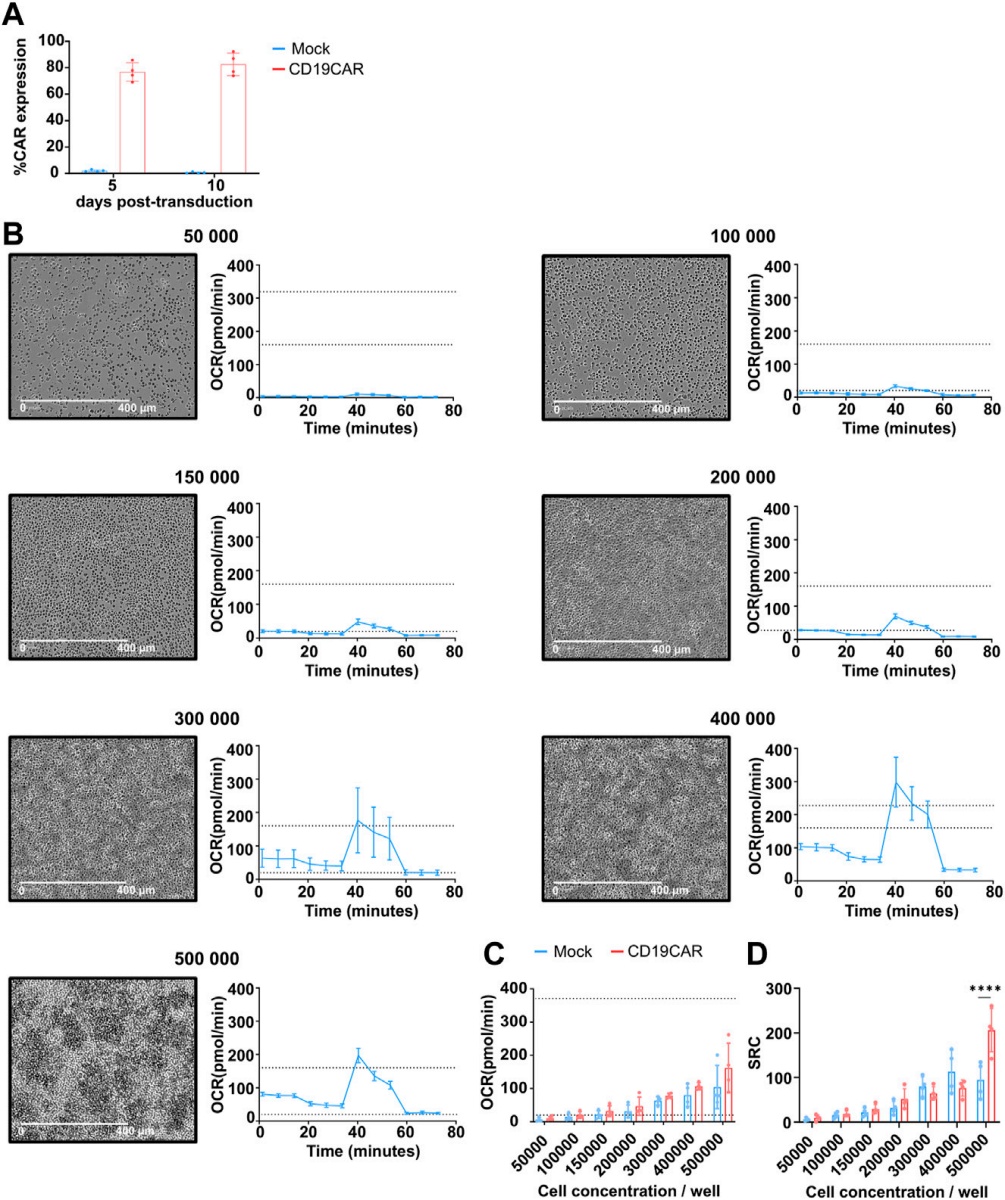
Optimization of cell concentration on mock T cells. **(A)** Quantification of CD19CAR expression in primary T cells at 5 and 10 days following transduction. **(B)** Cell distribution microscopy (left, scale bar: × 400 pm, × 10 objective) and respiration profile (right, dotted lines are the basal OCR range, 20 and 160 pmol/min) for the indicated cell concentration, these data were obtained from a single donor. **(C)** Basal OCR obtained for each cell concentration of mock and CD19CAR T cells. These results are presented as mean ± SD; (*n* = 4 donors). **(D)** SRC was calculated from the average maximal OCR—the average basal OCR for each cell concentration of mock and CD19CAR T cells. The results are mean ± SD; (*n* = 4 donors) and comparision between groups was performed with Two-way ANOVA test, ****p < 0.0001 (Only statistically different conditions are indicated on the graph).

The cell concentration is an important factor when studying metabolism. Indeed, too many cells seeded in a non-even monolayer could cause a hypoxic environment and thus skew the measurement of metabolic data. There are two ways to determine the optimal cell density. The first relies on the visual cell density, which is recommended to be between 50% and 90% confluence and uniformly distributed as a cell monolayer. The second way is based on the values obtained from the basal OCR which can confirm the optimal cell density and enhance the accuracy of the data. When using a Seahorse XF96 instrument, the manufacturer recommends the basal OCR to be between 20 and 160 pmol/min (Figure 1A). In order to find the optimal cell concentration for CAR T cell, we varied the number of cells per well from 50,000 to 500,000 within the same run. In addition, since the variability from donor to donor may influence the outcome (see below), these experiments were performed with T cells isolated from 4 different donors transduced or not with a CD19CAR. The expression of CD19CAR was confirmed by staining with CD19 chimaera (Figure 2A).

The cell density was assessed by imaging the 96-well plate using microscopy to visualize the distribution of cells in each well. As shown, a rough estimate of cell confluency resulted in 50%–90% confluence when 100,000 to 300,000 T cells were seeded per well (Figure 2B). Outside of these concentrations, the T cell density appeared suboptimal. Of note, T cell aggregates were detected at concentrations higher than 300,000 cells per well, which might greatly affects the proper detection of the metabolic influx as well as reproducibility of the data. This was already reported for metabolic measurement of stem cells (Zhang et al., 2012) but was not always respected in published CAR T cell experiment (Forcados et al., 2022).

The basal OCR was run in parallel and representative profiles are presented (Figure 2B, right). As expected, the OCR measures increased together with the cell concentration until the monolayer was disrupted. Data obtained from 4 donors revealed an average basal OCR between 20 and 160 pmol/min for 150,000 to 500,000 cells per well for both mock and CD19CAR T cells (Figure 2C). As shown, T cell concentrations lower or equal to 150,000 and higher than 300,000 per well provided the most heterogeneous detection of basal OCR confirming the need of maintaining a proper monolayer to further reduce donor variations. From these data, we concluded that the optimal range to compare mock and CD19CAR T cells should be 200,000 cells per well. Remarkably, we observed that the T cell spare respiratory capacity (SRC, see Figure 2D), corresponding to the maximum energy a cell can provide, also varied with cell concentration. The low SRC value detected at low cellular concentration was connected to the low detection in basal and maximal OCR. This low OCR was in turn linked to the limited response these few cells could evoke. Accordingly, SRC interpretation seems influenced by the sensitivity of the measure hence rationalizing the use of a proper seeding density to ensure precise and reproducible conditions but also highlight on how out-of-range conditions could lead to misinterpretation (Figure 2D; above 300,000 cells/well). Similar observations were made when comparing mock and CAR T cells at a concentration of 500,000 cells per well; a difference in SRC could be detected, however, this difference should not be taken in account due to the over-confluence of the cells (Figures 2C, D).

### 3.2 Optimization of drugs concentration

Another important factor is the evaluation of the concentrations of the compounds used in this assay. Indeed, an excessive or limited concentration could induce a total or partial inhibition, respectively, which in turn could affect the interpretation of the experiment. As previously mentioned, we here used the Mito Stress kit to measure mitochondrial respiration in CAR T cells using oxidative pathway inhibitors. This kit provides data on OCR as well as ECAR. It is based on the sequential addition of 3 compounds: Oligomycin, Carbonyl cyanide-p-trifluoromethoxyphenylhydrazone (FCCP), and Rote-none/Antimycin A.

**FIGURE 3:**
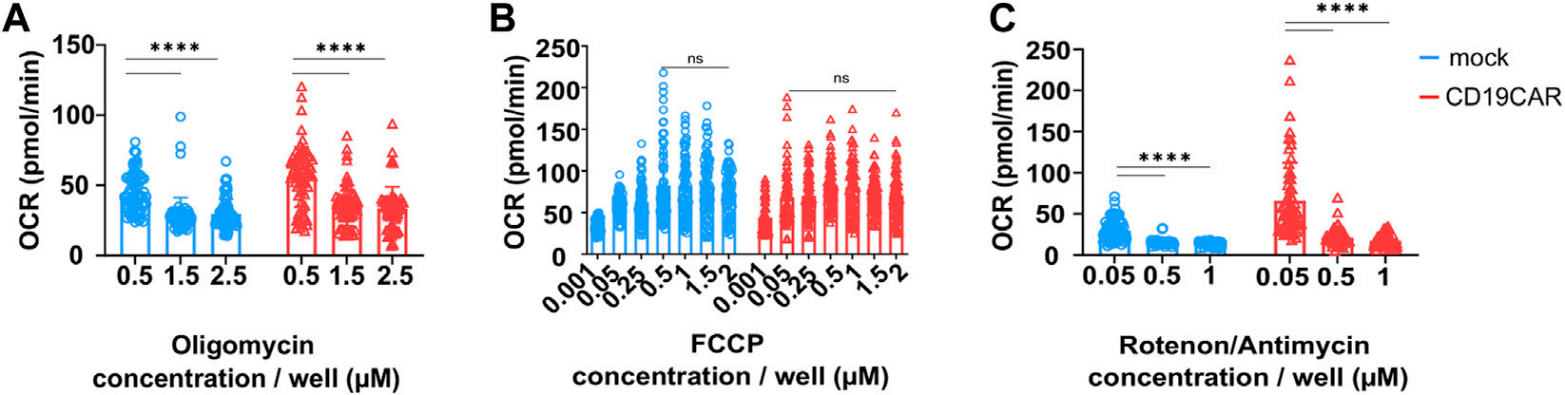
Optimization of drug concentration. Indicated T cells were seeded at 200,000 cells/well and their respiration was tested with varying **(A)** Oligomycin, **(B)** FCCP, and **(C)** Rotenone/Antimycin concentrations. The OCR values plotted on the graphs (left) correspond to the OCR counted after the addition of the indicated drug, results are mean ± SD, N = 4 donors in sextuplicate (*n* = 6) and comparison between groups was performed with Two-way ANOVA test, ****p < 0.0001, ns; no significance.

Oligomycin is an inhibitor of the ATP synthase which reduces mitochondrial respiration related to the production of cellular ATP (Huijing and Slater, 1961) (Figure 1). We varied the concentration of Oligomycin to find out the optimize concentration to use for T cells. We evaluated the Oligomycin concentrations recommended by the manufacturer, which were 0.5, 1.5, and 2.5 µM for a set concentration of 1 µM FCCP and 0.5 µM Rotenone/Antimycin A. The results showed that 0.5 µM was not sufficient to properly inhibit ATP synthase (Figure 3A). This incomplete inhibition influenced the measurement of maximal respiration. In contrast, both 1.5 and 2.5 µM appeared to provide the desired inhibition (Figure 3A). Thus, we propose the use 1.5 µM final concentration in CAR T cell studies.

The second inhibitor is FCCP, an uncoupler of the mitochondrial electron transport chain. Its addition results in enhancing OXPHOS and ATP synthesis to the maximum rate (Heytler and Prichard, 1962) (Figure 1). Basal and maximal respiration values allow to calculate the SRC, which describe the amount of ATP produced under stress or under increased energy demand. It is recommended to use FCCP at concentrations ranging from 0.15 to 2 µM. Based on these results we propose to use a concentration of 1 µM on CAR T cells (Figure 3B). Here Oligomycin and Rotenone/Antimycin A were used at 0.5 µM.

The third and last injection is a mix composed of Rotenone, a complex I inhibitor (Lindahl and Öberg, 1961), and Antimycin A, a complex III inhibitor, which blocks mitochondrial respiration (Kim et al., 1999) (Figure 1), and is recommended to be used at 0.5 µM. We have tested concentrations ranging from 0.05 to 1 µM. We observed that 0.05 µM was too low to induce a complete inhibition of mitochondrial respiration. However, 0.5 µM seemed sufficient to obtain a broad effect, similarly to a higher concentration of 1 µM. Therefore, the suggested concentration, 0.5 µM, appears ideal for CAR T cells (Figure 3C). These measures were performed with the previously defined concentrations of Oligomycin and FCCP.

### 3.3 Inter- and intra donor variations

**FIGURE 4:**
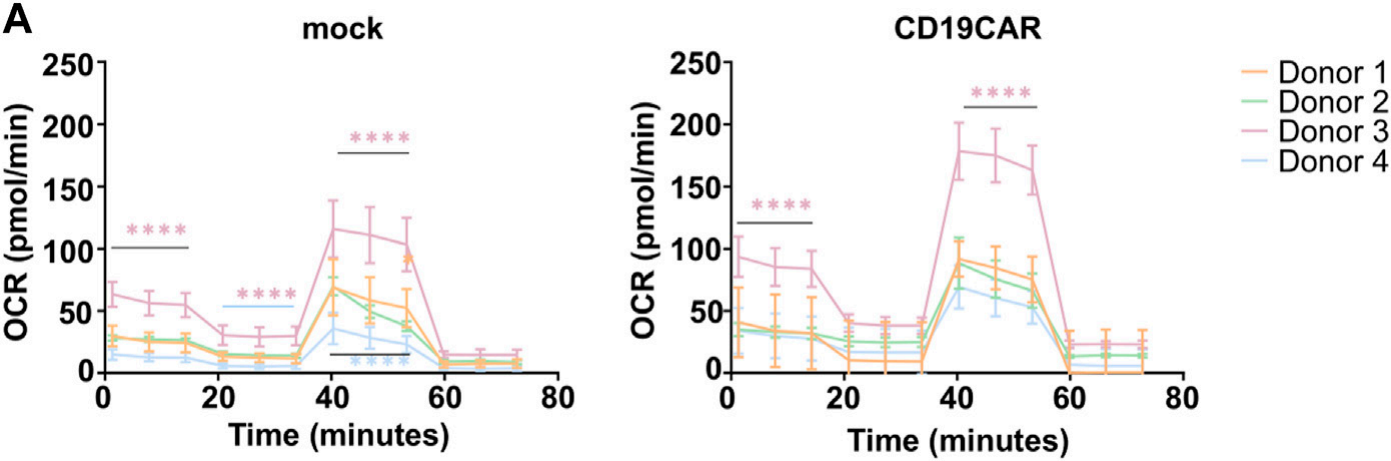
Reproducibility and variability. **(A)** OCR calculation plots of T cells isolated from four healthy donors (N = 4), mock and CD19CAR, run in sextuplicate (*n* = 6) in ideal conditions (200,000 cells, 1.5 µM of Oligomycin, 1 µM of FCCP, and 0.5 µM of Rotenone/Antimycin). The results are mean ± SD. Two-way ANOVA test was performed to compare groups. ****p < 0.0001.

Basal T cell metabolism and their metabolic response to various stimuli are sensitive to environmental factors, thus a small change in the conditions of cell isolation, or, as we have previously shown, in seeding of cells, might have an important impact on the calculated values and further interpretation of the data. There are large inter-donor variations with respect to CD4:CD8 T cell ratios which may affect the cytotoxic capacity of the T cell products. Their metabolic activity is similar and depends primarily on their activation status (Michalek et al., 2011; MacIver et al., 2013). Furthermore, different metabolic pathways are activated depending on the particular T cell subsets and their differentiation stage, which represents a serious issue when dealing, as we presently are, with a mixed T cell population. This is evidenced by the difference in antigenic response depending on the level of Oxygen (Maciolek et al., 2014; Kouidhi et al., 2017). In addition, cell culture conditions such as T cell expansion protocols can greatly influence the basal metabolic state of the cells (Forsdyke, 2020; Jenkins et al., 2021). In order to minimize the occurrence of false read-out and misinterpretation, one should measure each of the conditions in a minimum of six replicates. As shown in Figure 4, even in a controlled environment, the inter-donor variations are important, precluding the validity of data from one donor T cells as too often reported. Accordingly, inter-donor variability must be taken in account, as exemplified in Figure 4 where donor 3 showed a different respiration profile than the others. Since this type of variation cannot be avoided, one should aim at remaining as representative as possible, and thus work with T cells isolated from at least 4 different donors.

### 3.4 Metabolism under stimulation

**FIGURE 5:**
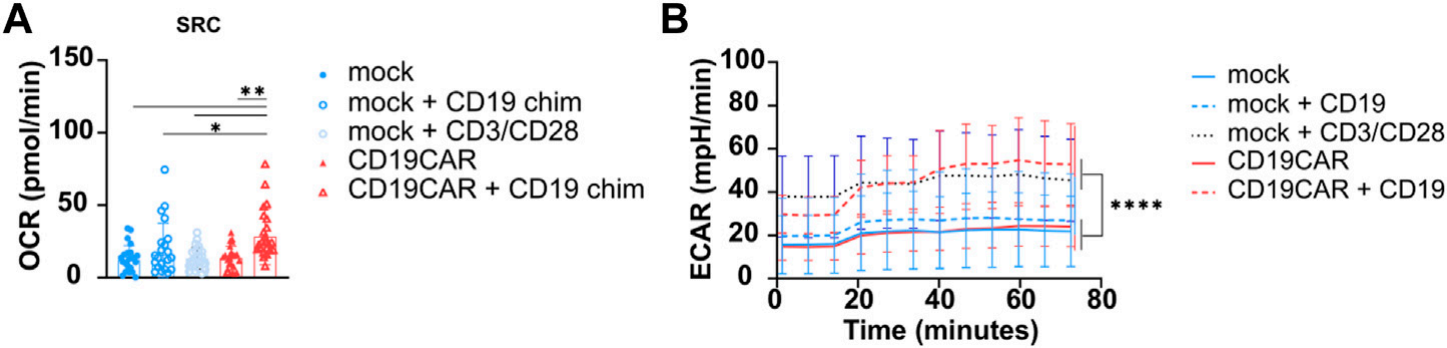
Stimulation of CD19CAR T cell. **(A)** Increased SRC of stimulated CD19CAR T cells. SRC was calculated from the average maximal OCR—the average basal OCR for each cell concentration of mock and CD19CAR T cells. **(B)** Increased ECAR for stimulated T cells (mock and CD19CAR). The experiments were run in optimal conditions as defined in the present report. The results are represented as mean ± SD; N = 4 donors in sextuplicate (*n* = 6); One-way ANOVA with multiple comparisons was performed to compare groups. *p < 0.1, **p < 0.01.

As previously mentioned, using the Seahorse XF96 analyzer, we observed a similar SRC for mock T cells and CD19CAR T cells without stimulation, using four donors under previously defined optimal conditions. After general (mock) or antigen-specific T cell stimulation (CD19CAR), using anti-CD3/CD28 or CD19, respectively, an increase in SRC was observed (Figure 5A). Thus, this assay is sensitive enough to detect CAR dependent rewiring of T cell stimulation and demonstrated that CD19CAR T cells use the mitochondrial pathway to produce energy when specifically stimulated. Thus, this assay could be exploited as a platform to screen different CAR constructs (based on signaling domain compositions and/or targeting units) in order to isolate the most metabolically favourable constructs. Additionally, it can also be used to screen CAR T cells generated by different *in vitro* expansion protocols to select for the one providing the most optimal metabolic profile.

In parallel, the glycolytic influx was also analyzed through the ECAR and mock T cells simulated with CD3/CD28 beads presented a higher ECAR compared to unstimulated T cells, confirming that T cell activation involves glycolytic pathways releasing protons in the milieu (Figure 5B). Importantly, we also noticed that CD19-stimulated cells displayed a higher ECAR (similar to stimulated mock T cells) than un-stimulated CAR T cells and mock T cells (Figure 5B). The increased glycolysis is in agreement with the CAR-expressing T cells becoming activated effector T cells ready to kill their targets. It should be highlighted that different CAR constructs containing different scFv or targeting units can influence the metabolic profile of CAR T cells (Kawalekar et al., 2016).

Together these results, performed in optimized conditions, showed a greater dependency of mock and CD19CAR T cells on glycolysis upon stimulation due to proliferation.

## 4 Conclusion

The assessment of CAR T cell metabolic state is of high importance to help predict the therapeutic potential of a cell therapy product. Indeed, investigators have shown that the TME has a strong negative influence on the efficacy of infused CAR T cells and that the co-transduction of CARs and specific metabolic enzymes improve the fitness of the cells and consequently the anti-cancer response. Recent reports have shown that CAR T cells need to have a longer persistence, with pronounced tumour infiltration potential and efficient killing function—all of those are driven by a defined and optimal T cell metabolism—in order to tackle the hardest-to-treat cancers such as the solid cancers and to further prevent the occurrence of relapses (Rostamian et al., 2021; Zhang et al., 2021). In agreement with Mercier-Letondal, P et al. a Seahorse-mediated assessment of T cells metabolic influx should be incorporated in the potency assays because it provides sound and instantly readable metabolic data that strengthen the characterization of CAR T cells (Mercier-Letondal et al., 2021). Still, to our knowledge, no optimization studies had been properly conducted on primary human CAR T cells and the increasing use of Seahorse urges for a harmonization of the protocols.

Here we described the optimized conditions to evaluate the metabolism of CAR T cells using a Sea-horse XF96 analyzer. This report complements our previous review discussing Seahorse use in the context of CAR T cell studies (Forcados et al., 2022), where we noticed that although successful results were often reported (van der Windt et al., 2016; Milam et al., 2020; Mercier-Letondal et al., 2021; Kong et al., 2022), the conditions between labs dramatically varied. To our knowledge, no optimization studies had been properly conducted on primary human CAR T cells and the increasing use of Seahorse urges for a harmonization of the protocols.

We showed that with or without activation of CAR, it is possible to calculate OCR and ECAR values after some optimization: we recommend the use of a concentration of 200,000 T cells per well for optimal and unbiased OCR calculations. We addressed the evaluation of the drug concentrations and observed that concentrations of 1.5 µM of Oligomycin, 1 µM of FCCP and 0.5 µM of Rote-none/Antimycin A provided the most reliable read-out. Finally, the less controllable factors were the inter-donor variation which dramatically influences the measures and the interpretation of the results. This can therefore have a large impact when low metabolic variations are expected, for instance, during the evaluation of new CAR candidates. Therefore, published experiments that intend to highlight a specific metabolic phenotype for a new CAR molecule should be reproduced in at least 4 donors to be valid. Besides, the intra-experimental variation and the occurrence of technical errors leading to misinterpretation of an effect can be avoided by respecting the use of sextuplicate for each condition.

The limitations of our study are the restricted use to one CAR construct. We cannot exclude that CAR with a different avidity for the same antigen or a different specificity might induce alternative type of metabolic changes. In addition, the influence of the CAR scaffold (hinge, TM, signalling domains) might also affect respiration capacity and this should also be assessed, as in Kawalekar et al., (2016). Another limitation lies in donor variability, due to the lack of information concerning his/her life style and health statute which can greatly affect the metabolism of his/her T cells. A standardized collection (age, sex, BMI, etc.) of PBMCs might reduce this variability when testing CAR modified cells. We hope that this work comparing the effect of different parameters will pave the way to a harmonized Seahorse protocol adapted to the metabolic evaluation of CAR T cells.

## Data Availability

The original contributions presented in the study are included in the article/Supplementary Material, further inquiries can be directed to the corresponding author.
